# Nano-photosensitizer based on layered double hydroxide and isophthalic acid for singlet oxygenation and photodynamic therapy

**DOI:** 10.1038/s41467-018-05223-3

**Published:** 2018-07-18

**Authors:** Rui Gao, Xuan Mei, Dongpeng Yan, Ruizheng Liang, Min Wei

**Affiliations:** 1 State Key Laboratory of Chemical Resource Engineering, Advanced Innovation Center for Soft Matter Science and Engineering, Beijing University of Chemical Technology, Beijing, 100029 China; 2 Beijing Key Laboratory of Energy Conversion and Storage Materials, College of Chemistry, Beijing Normal University, Beijing, 100875 China

## Abstract

Singlet oxygen has won a great deal of attention to catalysis and biological studies due to its strong oxidizing properties. However, the photosensitizers which require for the generation of singlet oxygen remain inadequate because of their lack of long-wavelength absorption, weak hydrophilicity, and poor biocompatibility. Here, we develop near-infrared laser activated supramolecular photosensitizers (isophthalic acid/layered double hydroxide nanohybrids) for efficient two-photon photodynamic therapy. The singlet oxygen quantum yield of nanohybrid is up to 0.74. Critically, in vitro tests verify the superior anti-cancer properties of nanohybrid with an IC_50_ determine to be 0.153 μg mL^−1^. The nanohybrids take advantage of the superior tissue penetration of 808 nm laser irradiation and exhibit a dramatically strong ability to ablate tumors in vivo, with extremely low toxicity. This work provides the proof of concept that ultralong-lived triplet excitons can function as two-photon-activated photosensitizers for an effective singlet oxygen generation.

## Abstract (web summary)

Usually, several components are needed for efficient 2-photon photodynamic therapy (PDT). Here, the authors sandwiched carboxylic acids between layered double hydroxide nanosheets to obtain a single-handed biocompatible photosensitizer that generates singlet oxygen in high quantum yield.

## Introduction

Singlet oxygen (^1^O_2_) has been demonstrated to have a relatively long lifetime and is able to persist in the gas phase for over an hour and for 10^−6^to 10^−3^ second in solution^1,2^. Among different kinds of reactive oxygen species (ROS), ^1^O_2_ presents promising applications in sewage treatment, photooxidation catalysis, and photodynamic therapy (PDT), as a result of its high reactivity and oxidizing capability^3–7^. Unfortunately, because of the spin-flip restriction between the singlet excited state (S) and the triplet state (T), the spontaneous conversion process that occurs from O_2_ to ^1^O_2_ is considered to be almost forbidden in the case of normal conditions. Therefore, photosensitizers are typically required to achieve effective ^1^O_2_ generation^8^. Over the past decades, a number of photosensitizers, including organic dyes, metal–organic complexes (i.e., porphyrins and phthalocyanine), and noble metal nanoparticles have been exploited in the field^4^. However, the majority of these photosensitizers possess some drawbacks, including weak hydrophilicity and dark toxicity. These characteristics lead to unsatisfactory ^1^O_2_ efficiency and therefore limit their use in further applications^9–12^. In recent years, the use of graphene, silicon, and black phosphorus was proposed for the generation of ^1^O_2_. Thus, preliminary studies were carried out on the applications of these systems in both photocatalysis and PDT. However, due to the fact that they lack the long-wavelength absorption band (typically below 700 nm), their photodynamic applications remain restricted by the insufficient ability to penetrate tissue and the potential photo-induced damage^9–12^. As of recently, the use of near-infrared (NIR) activated PDT technology has been considered a promising method to increase treatment depth, and also exhibits great potential for the use in medical applications. Most notably, two-photon PDT technology currently has both better penetration depth and spatial selectivity^13–19^. However, it remains difficult to synthesize NIR activated photosensitizers, and they suffer from a relatively low two-photon absorption efficiency^13–15^. Thus, it remains a challenging goal to generate high-efficiency PDT photosensitizers with simultaneously NIR excitation and excellent ^1^O_2_ generation.

Over the past few years, studies have demonstrated that certain organic aromatic compounds that exist in the crystalline state could exhibit a long-lived triplet-exciton emission under ambient conditions. This is also referred to as room temperature phosphorescence (RTP), which has peaked a particular interest in the fields of both sensor and optical imaging^20–24^. The excited triplet state lifetimes (millisecond to a second range) are typically greater than that of conventional photosensitizers (microsecond to millisecond range). Therefore, we considered the idea that the suitable energy level and ultralong-lived triplet exciton of RTP materials could function as a potential mechanism to greatly extend the collisional time and energy transfer to surrounding O_2_ for the purpose of ^1^O_2_ generation. In this study, we have achieved NIR-induced production of ultralong-lived triplet exciton through the generation of supramolecular photosensitizers with different aromatic RTP molecules that are confined in a 2D matrix. Layered double hydroxides (LDHs) were chosen as the 2D matrix due to their versatility in elemental composition, chemical stability, photostability, and superior biocompatibility^25,26^. These properties have also been studied in the fields of drug/gene delivery and bioinorganic composite materials^26–37^. Following fixation into the 2D nanogallery of LDHs, the *n* → π* transition of the carboxylic group was found to be favorable to the enhancement of spin–orbit coupling and long-lived triplet excited states^22,38–40^. In addition, the rigid and crystalline LDH sheets provide an interface- and space-confined microenvironment for RTP chromophores. This favors the formation of an orderly arrangement of phosphor aggregation with a prolonged triplet excited lifetime^40–46^. Finally, the LDHs matrix was found to promote the hydrophilicity and biocompatibility of the RTP molecule, resulting in enhanced drug permeability and retention^47^.

**Fig. 1:**
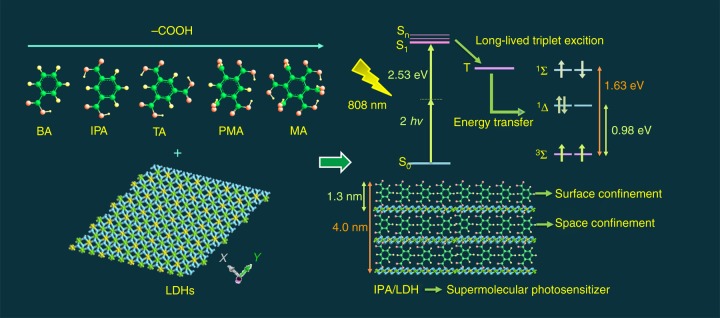
Illustration of nanohybrids as two-photon photosensitizers for the ^1^O_2_ generation. Schematic representation of the LDH host and five aromatic RTP guest species, as well as the 2D confined long-lived triplet excitons can function as a photosensitizing means to achieve efficient ^1^O_2_ generation under an 808 nm NIR laser

Here we show that the 2D confined long-lived triplet exciton can function as a photosensitizing means to achieve efficient ^1^O_2_ generation under an 808 nm NIR laser (Fig. 1). The two-photon induced ^1^O_2_ quantum yield generated from LDH-based self-assembles is observed to reach 0.74, a value greater than the majority of as-reported photosentizers to date. In addition, the PDT is demonstrated based on in vitro tests, which exhibits an excellent performance, and superior biocompatibility. In vivo therapy studies are also found to exhibit a dramatically strong ability to complete tumor ablation, and toxicity studies with a large LDH nanohybrid dose show hardly any in vivo toxicity. Therefore, this work demonstrates a successful paradigm of the effective ^1^O_2_ generation via NIR excitation based on a self-assembled photosensitizer, which exhibits promising applications for PDT cancer therapy.

## Results

### Structural and optical characterization

**Fig. 2:**
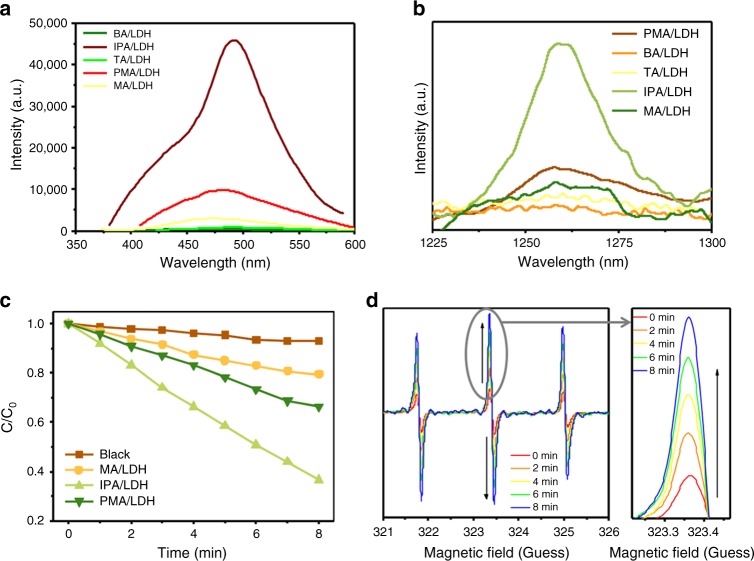
Optical characterization and identification of ^1^O_2_ generation. **a** Photoemission spectra of BA/LDH, IPA/LDH, TA/LDH, PMA/LDH, and MA/LDH nanohybrids TPA excitation by an 808 nm laser at the same power. **b** ^1^O_2_ emission at around 1270 nm induced by BA/LDH, IPA/LDH, TA/LDH, PMA/LDH, and MA/LDH nanohybrids in water. **c** Normalized absorbance of DPBF in the presence of IPA/LDH, PMA/LDH, and MA/LDH nanohybrids in air. **d** Time-dependent ESR spectra of IPA/LDH nanohybrids in the presence of TEMP

In order to achieve confined triplet exciton for high-efficiency ^1^O_2_ generation, a total of five different organic carboxylic molecules (namely benzoic acid (BA), isophthalic acid (IPA), trimesic acid (TA), pyromellitic acid (PMA), and mellitic acid (MA)) were assembled into the interlayer gallery of the Zn–Al–LDH host utilizing a co-precipitation method (Fig. 1). Powder XRD patterns (Supplementary Figure 1) demonstrate that in each case, all sample reflections can be indexed to a rhombohedral lattice. This is assigned to the 3R-type LDH structure description. Upon UV irradiation at 320 nm, we observed intense blue emissions from the BA/LDH, IPA/LDH, TA/LDH, PMA/LDH, and MA/LDH nanohybrids (Supplementary Figure 2 orange lines). Following removal of the UV source, the emission color was observed to change to green (Supplementary Figure 2 green lines). Time-resolved luminescence decay data demonstrated that all of these LDH hybrids exhibited long RTP lifetimes (29 ms, 1.17 s, 40 ms, 67 ms, and 117 ms, Supplementary Figure 3). This is suggestive of an effective production of long-lived triplet exciton. The corresponding RTP quantum yields of BA/LDH, IPA/LDH, TA/LDH, PMA/LDH, and MA/LDH nanohybrids were measured as 0.06%, 3.03%, 0.71%, 1.69%, and 1.24%, respectively. Moreover, in regard to the down-conversion (single-photon) triplet exciton generation, we observed that the long-lived triplet states were also produced through a two-photon excited process. Upon excitation with an 808 nm femtosecond laser, the BA/LDH, IPA/LDH, TA/LDH, PMA/LDH, and MA/LDH systems exhibited low-wavelength phosphorescence, with emission peaks in very close proximity to those excited by 320 nm irradiation (Supplementary Figure 4). Furthermore, the plots of log(intensity) vs. log(incident energy) for LDH nanohybrids (Supplementary Figure 5) exhibited a relatively good linear relationship, with slopes calculated to be close to 2. This suggests that the process is a two-photon mechanism. In addition, from our studies, it was clear that the emission intensities of the IPA/LDH, PMA/LDH, and MA/LDH systems were much higher than those of BA/LDH and TA/LDH (Fig. 2a) through the two-photon-excited process. This is consistent with their high time-resolved luminescence decay and triplet-exciton quantum yields. It is currently established that ^1^O_2_ is able to undergo radiative decay at ~1270 nm. To choose suitable supramolecular photosensitizers for the study of PDT, the ^1^O_2_ emission at around 1270 nm of LDH nanohybrids was studied (Fig. 2b), and it is obviously that the emission intensity at around 1270 nm of the IPA/LDH system was much higher than those of others. Because of the better two-photon excited process and ^1^O_2_ emission performance of IPA/LDH, PMA/LDH, and MA/LDH, these systems were chosen as the potential supramolecular photosensitizer for the next study.

Typically, the molecular oxygen activation can also be examined by using 1,3-diphenylisobenzofuran (DPBF) as a probe molecule, since DPBF can react with ^1^O_2_ undergoing an irreversible Diels–Alder 1,4-cycloaddition, inducing a decrease of the absorption intensity at about 410 nm. Here, the efficiency of ^1^O_2_ production was evaluated for IPA/LDH, PMA/LDH, and MA/LDH nanohybrids using DPBF as a probe. As depicted in Supplementary Figure 6, the absorption intensity of DPBF at 410 nm in the presence of IPA/LDH, PMA/LDH, and MA/LDH samples was observed to gradually decrease with increasing NIR light irradiation time (808 nm laser). The slope of the curve was observed to be proportional to the efficiency of ^1^O_2_ production. The IPA/LDH sample provides a steeper decline compared to PMA/LDH and MA/LDH within an 8 min NIR irradiation, indicating a stronger capability of ^1^O_2_ production (Fig. 2c). Electron spin resonance (ESR) spectrum is thought to provide the most direct evidence for the identification of the generated ROS species. In order to further confirm ^1^O_2_ generation by IPA/LDH, PMA/LDH, and MA/LDH nanohybrids, we utilized 2,2,6,6-tetramethylpiperidine (TEMP) as the ^1^O_2_ trapping agent in order to study the system using ESR under 808 nm laser irradiation. As depicted in Fig. 2d and Supplementary Figure 7, the time-dependent ESR signals for IPA/LDH, PMA/LDH, and MA/LDH nanohybrids clearly displayed a 1:1:1 triplet signal characteristic. This is consistent with those for 2,2,6,6-tetramethylpiperidine-N-oxyl (TEMPO). These results confirm that ^1^O_2_ is generated by IPA/LDH, PMA/LDH, and MA/LDH, in which the time-dependent signal intensity of TEMPO for IPA/LDH was also found to be significantly higher than that of PMA/LDH and MA/LDH.

**Fig. 3:**
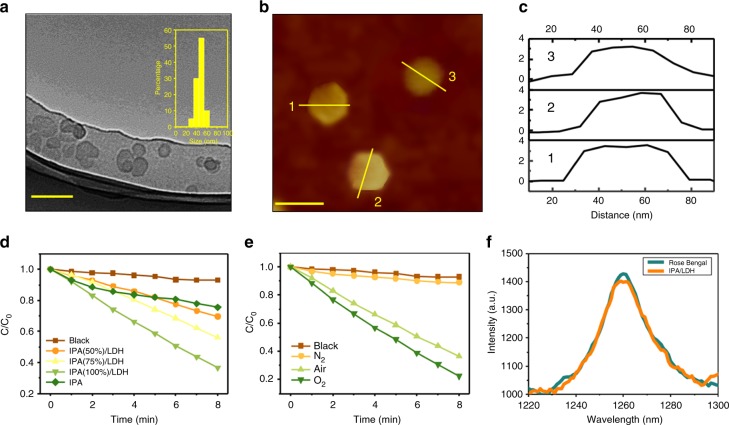
Structural characterization and evaluation of ^1^O_2_ production efficiency. **a** TEM image, Scale bar=100 nm. **b** AFM image, Scale bar=50 nm. **c** Height curves for IPA/LDH. **d** Normalized absorbance of DPBF in the presence of IPA, IPA(50%)/LDH, IPA(75%)/LDH, and IPA(100%)/LDH nanohybrids in air. **e** Normalized absorbance of DPBF in the presence of IPA/LDH under different atmosphere. **f** ^1^O_2_ emission at around 1270 nm induced by commercial Rose Bengal and IPA/LDH

Moreover, the morphology of IPA/LDH was studied. Transmission electron microscopy imaging (TEM, Fig. 3a) revealed that IPA/LDH consists of pseudo-hexagonal nanosheets with a diameter distribution of ca. 50 nm. The typical tapping mode AFM image for IPA/LDH nanohybrids (Fig. 3b, c) also demonstrates ~50 nm diameter and ~4 nm thickness of IPA/LDH, confirming the formation of IPA/LDH ultrathin nanosheets. In addition, based on the basic spacing of IPA/LDH (1.3 nm) from the XRD result and the ~4 nm thickness, the IPA/LDH supramolecular structure can be determined. In cases where the IPA monolayer is anchored at both the interface and interlayer regions (shown in Fig. 1), this further suggests that both the interface-confined and interlayer-confined effects occur for the IPA/LDH nanosheets. These dual confinements could greatly restrict both the IPA phosphor translation and rotation, thus favoring the enhancement of triplet lifetimes of IPA/LDH compared with pure IPA. To study the colloid stability, the size distribution and polydispersity of IPA/LDH nanohybrids were also measured by dynamic light scattering in water, PBS and cell culture medium (DMEM) for 14 days. As shown in Supplementary Figure 8, IPA/LDH exhibits equivalent hydrodynamic diameter distribution in day 1 and day 14 with a little increased polydispersity indexes (PDI). In addition, the insert images in Supplementary Figure 8a and 8c exhibit obvious Tyndall effect for IPA/LDH dispersed in water, PBS and DMEM, indicating the formation of uniform and stable colloid without aggregation for 14 days.

In order to study the effect of different intercalation contents of IPA on ^1^O_2_ production, we co-assembled IPA and SDS into the LDH interlayer region. Here, SDS functions as a dispersant for controlling the IPA loading and distribution. The XRD patterns of IPA(*x*%)/LDH samples are depicted in Supplementary Figure 9. In each case, the XRD pattern was found to exhibit characteristic reflections of the LDH layered structure with a series of (00*l*) peaks that appear as narrow, strong lines at low angles. This indicates that the guests have been successfully intercalated into the LDH gallery for the production of the supramolecular structure. We next measured the ^1^O_2_ production efficiency of IPA(*x*%)/LDH. The ^1^O_2_ emission at around 1270 nm of IPA(*x*%)/LDH is shown in Supplementary Figure 10: the IPA(100%)/LDH shows the highest emission intensity at around 1270 nm, which is much higher than those of the others. Supplementary Figure 11 demonstrates the absorption intensity of DPBF at 410 nm in the presence of numerous samples as a function of exposure time with 808 nm light. IPA(100%)/LDH demonstrates the fastest decrease in absorption intensity of DPBF relative to pristine IPA and other IPA(*x*%)/LDH at the equivalent IPA concentration. This indicates that the sample of IPA(100%)/LDH displays the strongest ^1^O_2_ production efficiency (Fig. 3d). This behavior is thought to be related to the formation of molecular aggregates and the ordered arrangement of IPA within the confined LDH matrix. This would be expected to promote the triplet excitation, thereby dramatically enhancing ^1^O_2_ generation. This aggregation-induced triplet enhancement may function to overcome traditional photosensitizer systems, including phthalocyanine (Pc), with the aggregation-induced quenching of the triplet yield and concomitant ^1^O_2_ production. In addition, in order to exclude the possible direct reaction between DPBF and photo-excited IPA/LDH nanohybrids, DPBF photolysis was carried out under a continuous O_2_/N_2_ purge, respectively (Fig. 3e and Supplementary Figure 12). The absorption of DPBF was maintained nearly unchanged under N_2_ condition, while it was observed to dramatically decrease under O_2_ condition. The oxygen content-dependent characteristic clearly indicates that ^1^O_2_ is generated under a photosensitizing process via energy transfer from its triplet to ground-state oxygen.

To evaluate of the ^1^O_2_ quantum yield, here, Rose Bengal (RB) was used as a standard photosensitizer, and the ^1^O_2_ quantum yield of IPA/LDH was calculated using the following formula: *Φ*_IPA/LDH_ = *Φ*_RB_ (*I*_IPA/LDH_/*I*_RB_). As shown in Fig. 3f, the integral areas of ^1^O_2_ luminescence that were produced by IPA/LDH nanohybrids and RB are ~124628 and 128482, respectively. These calculations are based on the value for *Φ*_RB_ = 0.76 in D_2_O, and *Φ*_IPA/LDH_ is estimated to be 0.74^48^. To the best of our knowledge, this value is greater than those of the majority of as-reported photosentizers. In order to confirm this claim, we further compared the *Φ* value of ZnPc and IPA/LDH. The value for *Φ*_ZnPc_ is 0.53 in EtOH^48^. This is lower than *Φ*_IPA/LDH_, which indicates the superior ^1^O_2_ generation ability of IPA/LDH. In addition, IPA/LDH exhibits an enhanced photostability relative to ZnPc under a simulated light source (100 mW cm^−2^, 30 min, Supplementary Figure 13). To characterize the two-photon excitation efficiency of IPA/LDH, its two-photon absorption (TPA) cross section was measured (Supplementary Figure 14). The measured TPA cross section is approximately >1000 gm from *λ* = 780 to 880 nm, which reaches 3000 gm at 860 nm. Such a large TPA cross section enables a great potential of the IPA/LDH for PDT.

### In vitro study with Hela cells

**Fig. 4:**
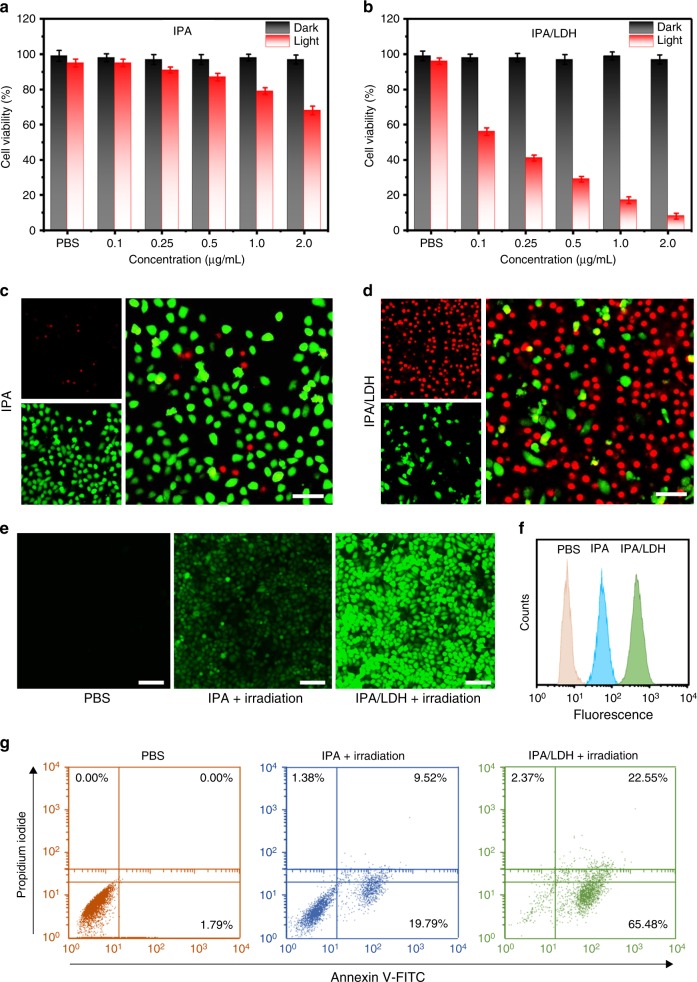
In vitro PDT effects of IPA/LDH with Hela cells. MTT assay of cell viabilities with and without irradiation following incubation with **a** IPA and **b** IPA/LDH, respectively. The corresponding Calcein-AM/PI staining images of cells treated with **c** IPA and **d** IPA/LDH following irradiation. Scale bar=50 nm. **e** Intracellular ROS production treated with different samples using DCFH-DA as a probe. Scale bar=100 nm. **f** Flow cytometry measurements of DCFH-DA signal for PBS (pink), IPA (blue), and IPA/LDH (green). **g** Flow cytometry measurement results of Annexin V-FITC/PI double stained cells treated with PBS, IPA, and IPA/LDH followed by laser irradiation. Error bars were defined as standard deviation, *n* *=* 3

To study the in vitro and in vivo behaviors of IPA/LDH, a near-infrared (NIR) dye, Cy5.5 was choose to label IPA/LDH^49,50^, which makes the nanoparticles to be readily detected by confocal laser fluorescence scanning microscopy (CLSM). For this purpose, Cy5.5-labeled IPA/LDH was prepared and showed stable fluorescence signal as Cy5.5 for 14 days (Supplementary Figure 15), promising its further application. As shown in Supplementary Figure 16, Hela cells incubated with free Cy5.5 (Supplementary Figure 16a) only exhibited weak fluorescence intensity from 1 h to 12 h while the fluorescence intensity increased obviously in the group of Cy5.5-IPA/LDH (Supplementary Figure 16b), indicating the greatly enhanced cellular uptake ability of Cy5.5-IPA/LDH. The fluorescence intensity of cells incubated with Cy5.5-IPA/LDH first increases sharply from 1 h to 12 h and further enhances to some extent from 12 h to 24 h, which shows the continuous cellular uptake of IPA/LDH within 24 h. Moreover, the decrease of fluorescence intensity from 24 h to 48 h may result from the cell metabolism of IPA/LDH. Due to the superior ^1^O_2_ generation properties, we studied the in vitro PDT efficiency and cytotoxicity of IPA/LDH against cancer cells. Hela cells were cultured in the presence of IPA and IPA/LDH with equivalent drug concentrations ranging from 0.1 to 2.0 μg mL^−1^ (prepared by diluting the storage solution with DMEM) for 24 h. Cells were then thoroughly washed using PBS, followed by irradiation in the presence or absence of NIR light (808 nm, 1.0 W cm^−2^, 5 min). The in vitro therapeutic efficacy was determined using the standard methyl thiazolyltetrazolium (MTT) assay. Prior to irradiation, IPA and IPA/LDH were found to have minimal effects on cell viability. Following further treated with concentrations as high as 500 μg mL^−1^, IPA showed some cytotoxicity while the cell viability of IPA/LDH was observed to be above 95% (Supplementary Figure 17), indicating superior biocompatibility. Following irradiation, anticancer effects were observed to be gradually enhanced with IPA and IPA/LDH treatments at increments from 0.1 to 2.0 μg mL^−1^ (Fig. 4a, b). The half maximal inhibitory concentrations (IC_50_) of IPA and IPA/LDH were determined to be 6.09 μg mL^−1^ and 0.153 μg mL^−1^, respectively. The enhanced efficiency of singlet oxygen production of IPA/LDH produces a greatly increased PDT activity. In order to visualize anticancer efficacy, Hela cells were treated with 2 μg mL^−1^ of IPA and IPA/LDH in the presence of NIR laser irradiation. Cells were then stained with calcein-AM and propidium iodide (Calcein-AM/PI) (Fig. 4c, d), to visualize living and dead cells, respectively. The results from these studies indicated a prominent cell death (strong PI signal) with IPA/LDH compared with only few cell mortalities with IPA. This is consistent with in vitro test results (Fig. 4a, b). Intracellular ROS production was determined using the cell permeable fluorescent dye, 2′,7′-dichlorodihydrofluorescein diacetate (DCFH-DA), which displays a green fluorescence signal following reaction with ^1^O_2_. As depicted in Fig. 4e, cells incubated with IPA in the presence of NIR irradiation exhibited only very minimal fluorescence, while cells treated with IPA/LDH under the same conditions exhibited a strong signal. These results indicate the superior ROS production. In addition, flow cytometry was utilized in order to quantify ^1^O_2_ production. The results depicted in Fig. 4f demonstrate that the IPA/LDH treated group (green peak) gave a promoted signal intensity relative to that of IPA (blue peak). We note that cell death was not induced by photothermal effects, as the temperature changed only slightly following 10 min of irradiation (Supplementary Figure 18). As shown in Supplementary Figure 19, the temperature elevation is only 3.23 ± 0.17 °C within 5 min (1.0 W cm^−2^, 808 nm) for cells incubated with IPA/LDH while it is almost the same for the group without IPA/LDH, indicating that the irradiation of the cell culture with 1.0 W cm^−2^ of 808 nm for 5 min does not obviously raise the temperature of the cells. In addition, cell apoptosis can be induced by intracellular ^1^O_2_ following IPA/LDH treatment with NIR irradiation, and data were analyzed using Annexin V-FITC/PI staining. As depicted in Fig. 4g, IPA treated cells with irradiation exhibited an apoptosis of 29.31%, while little apoptosis was observed to be induced in PBS treated cells. However, 88.03% of cells were observed to suffer from apoptosis following incubation with 2 μg mL^−1^ of IPA/LDH and irradiation. Thus, intracellular ROS generated by IPA/LDH under NIR light irradiation was found to clearly induce cell apoptosis and inhibit cell proliferation. We hypothesize that the increased PDT efficiency of IPA/LDH rather than pristine IPA is related to the following facts: (1) the confined triplet exciton in IPA/LDH exhibit an increased lifetime relative to IPA (289 ms);^20^ (2) 2D IPA/LDH ultrathin nanosheets would be expected to favor absorption and utilization of NIR light in order to fully interact with ground-state oxygen; (3) LDH nanosheets present a high hydrophilicity and biocompatibility for drug permeability and retention.

### In vivo biodistribution and pharmacokinetic profile

**Fig. 5:**
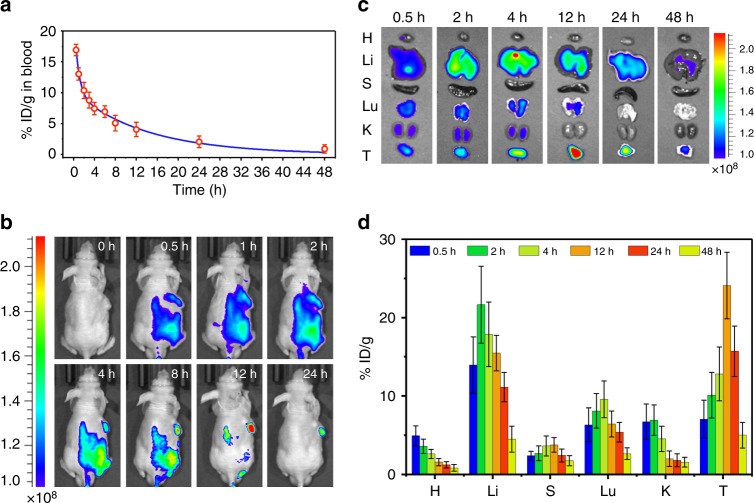
In vivo biodistribution and pharmacokinetic profile. **a** Blood circulation time of the Cy5.5-labeled IPA/LDH, the fluorescence intensity of Cy5.5 in the blood was determined over time after intravenous injection. **b** Time-dependent NIR fluorescence images of Hela tumor-bearing mice at 0 h, 0.5 h, 1 h, 2 h, 4 h, 8 h, 12 h, and 24 h post-injection. **c** Fluorescence images of heart (H), liver (Li), spleen (S), lung (Lu), kidney (K), and tumor (T) harvested from mice after intravenous injection of Cy5.5-labeled IPA/LDH at different time points. **d** Quantitative biodistribution analysis of the IPA/LDH in mice by measuring the Cy5.5 fluorescence intensity at different time points post-injection. Error bars were defined as standard deviation, *n* *=* 3

Since in vivo behavior is important for preclinical studies, the pharmacokinetics profile of the Cy5.5-labeled IPA/LDH was examined by determining the fluorometry in blood at different time intervals post-injection (at the drug dosage of 1 mg kg^−1^, 200 µL). The fitting result (Fig. 5a) shows the blood circulation of Cy5.5-labeled IPA/LDH obeys the typical two compartment model, in which the two blood circulation half-lives are 0.58 ± 0.08 h (distribution phase with a rapid decline) and 9.71 ± 0.93 h (elimination phase, the prolonged process for clearance), respectively. The volume of distribution (V) is measured to be 1.07 ± 0.24 mL and the area under curve (AUC) is 0.15 ± 0.07 mg h mL^−1^. Hence, the long blood circulation time facilitates the tumor accumulation of IPA/LDH ascribed to the enhanced permeability and retention (EPR) effect.

In addition, in vivo fluorescence imaging has been performed to determine the biodistribution of IPA/LDH in mice. As illustrated in Fig. 5b, the fluorescence signal of Cy5.5 appears in the tumor site at 0.5 h post-injection and gradually increases up to 12 h, revealing the effective tumor accumulation of IPA/LDH. The fluorescence intensity at tumor site decreases but remains strong after 24 h post-injection, which illustrates the excellent retention of IPA/LDH in the tumor. Figure 5c shows the ex vivo fluorescence images of main organs obtained at different time points, where the accumulation of IPA/LDH at tumor increases to the maximum at 12 h. For quantitative analysis, these tumors and organs were weighed and solubilized by lysis buffer followed by examining their fluorescence intensity to quantify the concentration of accumulated IPA/LDH. As shown in Fig. 5d, time-dependent distribution of IPA/LDH at tumor and various organs were determined and high levels of IPA/LDH were observed in the tumor, as well as reticuloendothelial systems (RES) such as liver, lung, and kidney, which is consistent with the ex vivo fluorescence images in Fig. 5c. Thus the drug-light interval was determined to be 12 h between tail vein injection and irradiation for further PDT treatment.

### In vivo cancer therapy

**Fig. 6:**
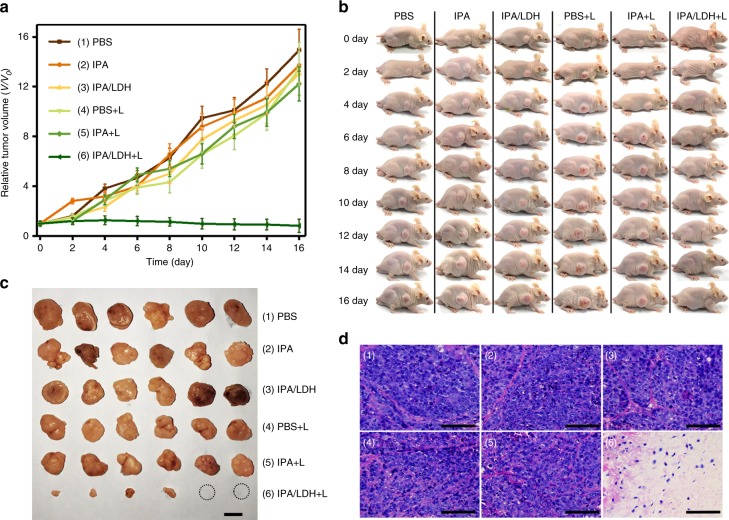
In vivo PDT effects of IPA/LDH. **a** Tumor growth curves of mice with the various treatments. Tumor sizes were normalized to their initial sizes (*n* = 6 per group). Error bars were defined as standard deviation, *n* *=* 6. **b** Representative photographs of mice with treatments at different days. NIR illumination was carried out for a 10 min exposure using an 808 nm laser at 0.7 W cm^−2^. **c** Corresponding images of tumors taken on day 16 treatment. Scale bar=1 cm. **d** H&E stained tumor tissue slices after 16 d treatments with (1) PBS, (2) IPA, (3) IPA/LDH, (4) PBS with irradiation, (5) IPA with irradiation, (6) IPA/LDH with irradiation. Scale bar=100 µm

In order to further reveal the potential in vivo PDT cancer therapy effects of IPA/LDH, we carried out an in vivo antitumor study on Balb/c nude mice bearing Hela tumors. Once the average tumor volume reached ~70 mm^3^, a total of 36 mice were divided into six groups: (1) PBS, (2) IPA, (3) IPA/LDH, (4) PBS with irradiation, (5) IPA with irradiation, and (6) IPA/LDH with irradiation. Treated mice were intravenously injected with a drug dosage of 1 mg kg^−1^ (from the storage solution diluted with PBS) followed by exposure to either an 808 nm laser (0.7 W cm^−2^) for 10 min or no irradiation at 12 h post injection. Tumor volume was monitored within 16 days (Fig. 6a). PBS, IPA, and IPA/LDH without irradiation, as well as saline and IPA with irradiation treatment groups, were found to exhibit negligible tumor inhibition. We note that tumor growth was significantly inhibited in the case of the IPA/LDH with irradiation treatment group. This indicates a remarkably enhanced in vivo PDT therapeutic effect. Digital photos of the mice growth tendency and corresponding excised tumors (Fig. 6b, c) visually demonstrate that the tumor size in mice treated with IPA/LDH with irradiation was significantly inhibited, while that of the other groups was greatly increased. It is also noted that the local temperature at tumor site increases within 3 °C for both the groups of PBS and IPA/LDH after irradiation (Supplementary Figure 20), indicating no obvious photothermal effect is produced by 808 nm NIR irradiation (0.7 W cm^−2^, 10 min). Moreover, compared with the control group without irradiation, the slice of skin overlying the tumor under irradiation also does not induce obvious skin damage, indicating the weak hyperthermic effect (Supplementary Figure 21). Hematoxylin and eosin (H&E) staining revealed that tumor tissue treated with IPA/LDH and NIR irradiation resulted in obvious necrosis, while control groups were observed to retain normal morphology (Fig. 6d). Moreover, no significant decrease was observed in the mice weight for each group during the treatment period, indicating no obvious side effects with these treatments (Supplementary Figure 22). Furthermore, we studied the in vivo toxicity of IPA/LDH treatment with intravenous injection of IPA/LDH (5 mg kg^−1^). Blood biochemistry (WBC, RBC, HGB, and PLT), as well as liver and kidney function markers (AST, ALT, BUN, and CRE) were all observed to be within their normal ranges following 1 day and 7 days, relative to healthy mice (Supplementary Figure 23). In addition, H&E analysis of major organs (heart, liver, spleen, lung, and kidney) (Supplementary Figure 24) showed no significant damage in the IPA/LDH and saline treatment groups, indicating a satisfactory safety profile of IPA/LDH used as a PDT cancer therapy.

## Discussion

In summary, we have successfully developed types of NIR-activated supramolecular photosensitizers for the highly efficient PDT through the assembly of different aromatic acids within LDH nanosheets. Both the space-confined and interface-confined microenvironments of LDH monolayers function to facilitate the two-photon-induced generation of ultralong-lived triplet exciton with second-scale lifetimes, and therefore promote ^1^O_2_ quantum yields as high as 0.74. Importantly, due to the combination of the effective ^1^O_2_ generation and 2D ultrathin nanosheets, in vitro tests were used to verify the superior anti-cancer properties of IPA/LDH with an IC_50_ of 0.153 μg mL^−1^, in addition to high biocompatibility. By taking advantage of the superior tissue penetration capabilities of the 808 nm laser irradiation, the as-prepared IPA/LDH ultrathin nanosheets exhibit dramatically strong tumor ablation capabilities in vivo, with extremely low toxicity. Compared with as-reported traditional systems which absorb two photons and then transfer the energy to a second photosensitizer with a long lived triplet state, this work may involve the case that two-photon PDT can be achieved using a single material. Therefore, this work not only puts forward an alternative method that the long-lived RTP materials can function as NIR-activated photosensitizers for the effective generation of ^1^O_2_, but it also offers a facile method for the development of promising nano-photosensitizers for future cancer therapeutic uses.

## Methods

### Synthesis of LDH nanohybrids

These hybrids were prepared by the coprecipitation method, in which the matched molar ratios of Zn^2+^/Al^3+^/IPA (or BA/TA/PMA/MA) were 2.0:1.0:1.0. In the case of IPA/Zn_2_Al-LDH, 100 mL solution containing 5.00 mmol Zn(NO_3_)_2_·6H_2_O and 2.50 mmol Al(NO_3_)_2_·9H_2_O was added dropwise to another 100 mL deionized water solution containing 15.00 mmol NaOH and 2.50 mmol IPA with vigorous agitation under a nitrogen flow. The pH value was adjusted to 7.0 (with 2.4 M NaOH solution) before heated to 60 °C for 24 h, The resulting solid production was filtered, and washed thoroughly with deionized water. The final nanohybrid was vacuum-dried at 45 °C for 24 h before use and a storage solution was also prepared by dispersion of IPA/LDH in PBS to form stabilized colloidal solution.

### Structural and morphology characterization

Rigaku Ultima-IV automated diffraction system with Cu K*α* radiation (*λ* *=* 1.5406 Å) were applied to collect the powder XRD patterns of all compounds in the power of 40 kV, 50 mA. Measurements were made in a 2*θ* range of 3−70° at room temperature with a step of 0.02° (2*θ*) and a counting time of 0.2 s per step. Inductively coupled plasma (ICP) atomic emission spectroscopy was performed on a Shimadzu ICPS-7500 instrument using solutions prepared by dissolving the samples in dilute nitric acid. The morphology of the simples was investigated by using high resolution transmission electron microscope (HRTEM JEOL, JEM-2100); the accelerating voltage was 200 kV. The thickness of the LDH nanosheets was measured using atomic force microscopy (AFM) software (Digital Instruments, Version 6.12). Electron spin resonance (ESR) measurements were carried out on a Bruker EMX plus model spectrometer operating at X-band frequencies (9.4 GHz) at room temperature.

### Optical properties

Photoluminescence including fluorescence and phosphorescence spectra, the time-resolved luminescence decay spectra of all the samples were collected on an Edinburgh FLS980 fluorescence spectrometer. The solution UV–vis absorption spectra were performed on a Shimadzu U-3000 spectrophotometer with the range from 300 to 800 nm in the slit width of 1.0 nm.

### Detection of singlet oxygen by DPBF

The generation of singlet oxygen of IPA/LDH, PMA/LDH, and MA/LDH were determined by using 1,3-diphenylisobenzofuran (DPBF) as a singlet oxygen probe. For every sample, a suspension was prepared by dispersing them in acetonitrile (equal concentration for IPA/PMA/MA: 5 μg mL^−1^) and stored in the dark. For example, A DPBF solution (15 μL, 59.5 μM) was added into 2 mL IPA/LDH suspension and mixed throughly, followed by irradiation with a Ti:sapphire femtosecond pulses laser (808 nm, 1.0 W cm^−2^). The decreased UV absorption intensity at 410 nm is proportional to the singlet oxygen production and the absorbance value was recorded per minute.

### Detection of ^1^O_2_ by ESR

The generation of ^1^O_2_ by IPA/LDH was confirmed by using 2,2,6,6-tetramethylpiperidine (TEMP) as trapping agent in electron spin resonance (ESR) measurement. Specifically, the ESR spectrum of IPA/LDH solution containing 97 μM TEMP was obtained with light irradiation. Moreover, PMA/LDH and MA/LDH group were set as the control group.

### Evaluation of the ^1^O_2_ quantum yield by detecting its emission

The ^1^O_2_ emission signal around 1270 nm was recorded by Edinburgh FLS980 fluorescence spectrometer with an excitation wavelength of 808 nm detector. As the standard photosensitizer, the ^1^O_2_ quantum yield of RB was measured to be 0.76 (*Φ*_RB_ = 0.76, in D_2_O). The ^1^O_2_ quantum yield of IPA/LDH was calculated by comparing the luminescence intensity toward that of RB. In detail, *Φ*_IPA/LDH_ = *Φ*_RB_(*I*_IPA/LDH_/*I*_RB_), where *I*_IPA_ and *I*_RB_ mean the PL peak areas of ^1^O_2_ produced by IPA/LDH and RB, respectively.

### Two-photon photophysical properties

To characterize the two-photon excitation efficiency of IPA/LDH, its TPA cross section was measured using two-photon-induced fluorescence method^51^. The TPA cross section spectra were obtained using a Tsunami-Spit-re-OPA-800C (Spectra physics) Ti-sapphire femtosecond laser in the range 760−880 nm. Spectra calibration was performed by comparing with the published Rhodamine B two-photon absorption spectrum^52^. The pulse duration is 120 fs and the peak power is 100 KW.

### In vitro studies on tumor cells

Hela cells were obtained from Institute of Basic Medical Sciences Chinese Academy of Medical Sciences (Beijing, China). Hela cells were grown in 25 cm^2^ cell-culture flask. After reaching 80–90% confluence, the Hela cells were detached by adding 1.0 mL trypsin (0.25%) for 2 min after washed with PBS. Hela cells were seeded into two 96-well plates in the density of 1×10^4^ cells per well. After 24 h, the cells were incubated with IPA/LDH suspension for another 24 h, one plate was irradiated with 808 nm (1.0 W cm^−2^) and another was stored in the dark. After incubated under 37 °C for another 6 h, the colorimetric 3-(4,5-dimethylthiazol-2-yl)-2,5-diphenyltetrazolium bromide (MTT) was used to determine the cell viability. In a typical cellular image experiment, cells were treated with 2 μg mL^−1^ of IPA/LDH, pristine IPA and blank, followed by incubating at 37 °C for 24 h. After washing with PBS for three times, cells were irradiated for 5 min (808 nm, 1.0 W cm^−2^), treated cells were stained with calceinacetoxymethyl ester (Calcein-AM) and propidium iodide (PI). Cells treated without irradiation were also studied as a reference sample.

### Cell apoptosis analysis

For the cell apoptosis analysis, Hela cells were seeded into 6-well plates in the density of 2 × 10^5^ per well and incubated for 24 h. Then the culture medium was replaced by 2 mL of DMEM containing IPA or IPA/LDH (in the concentration of 2 μg mL^−1^) and cells were incubated at 37 °C. After 24 h, cells were washed thoroughly with PBS and irradiated under 808 nm laser. After incubation for another 6 h, cells were collected and stained using an Annexin V-FITC Apoptosis Detection Kit purchased from Solarbio Life Sciences (Beijing, China), and examined with flow cytometry.

### Animal experiments

All animal procedures were performed in accordance with the National Institute of Health Guiding Principles for the Care and Use of Laboratory Animals and were approved by the Ethics Review Committee for Animal Experimentation of the Institute of Clinical Medicine, China-Japan Friendship Hospital, Beijing. Male Balb/c nude mice (Balb/c-nu, ∼20 g) were purchased from Beijing Vital River Laboratory Animal Technology Co., Ltd. The protocols of animal procedures have been approved by China-Japan friendship Hospital Animal Research Center. For every mouse, 100 μL phosphate buffered saline (PBS) with 5×10^6^ Hela cells suspended was subcutaneously implanted into the right shoulder. Mice were ready for further study when the volume of tumor was ~70 mm^3^.

### Pharmacokinetic and biodistribution analysis

Cy5.5 solution (0.1 mg mL^−1^) was added into IPA/LDH and stirred for 6 h. Excess dye was removed by centrifugation and washed thoroughly with water >5 times followed by resuspension in PBS.

To evaluate the pharmacokinetic properties, blood (10 µL) was collected from the tail veins of the Balb/c nude mice at certain time points after injection of the Cy5.5-labeled IPA/LDH. The concentration of Cy5.5-labeled IPA/LDH was determined by measuring the fluorescence intensity of blood sample dissolved in lysis buffer (1 mL). The blood sample from untreated mice was measured to be blank sample and subtracted during the concentration calculation through fluorescence intensities. A standard calibration curve was also determined by measuring a series concentration of samples. The pharmacokinetic parameters were determined using a Microsoft add-in tool, PKSolver^53^.

In vivo fluorescence imaging was performed on an IVIS-Spectrum imaging system under spectral unmixing mode and mice were anesthetized by isoflurane (1.0–2.0%) in oxygen. For the ex vivo fluorescence imaging, mice were sacrificed and major organs including the liver, spleen, kidney, heart, lung, and tumor were collected and imaged immediately. Afterwards, the tumor and organs were weighed and digested by a lysis buffer with grinding. The fluorescence intensities were measured by subtracting the background from untreated mice. The biodistribution was calculated and presented in the form of percentage of injected dose per gram of tissue (%ID g^−1^).

### In vivo photodynamics therapy

Mice were randomized into six groups of 6 animals per group: (Group i) 200 μL of saline injected intravenously without irradiation; (Group ii) 200 μL IPA (1 mg kg^−1^) injected intravenously without irradiation; (Group iii) 200 μL IPA/LDH (equivalent 1 mg kg^−1^ IPA) injected intravenously without irradiation; (Group iv) 200 μL of saline injected intravenously with irradiation at 0.7 W cm^−2^; (Group v) 200 μL of IPA injected intravenously with irradiation (0.7 W cm^−2^); (Group vi) 200 μL of IPA/LDH (equivalent 1 mg kg^−1^ IPA) injected intravenously with irradiation (0.7 W cm^−2^). All the irradiation was performed with 808 nm laser for 10 min. The tumor size was measured by a caliper every other day and calculated as the volume = (tumor length) × (tumor width)^2^ × 0.5. Relative tumor volume was calculated as *V*/*V*_0_ (*V*, *V*_0_ are the tumor volume measured at time *t* and *t*_0_, respectively).

### Histology examination and blood analysis

Mice of control and treated groups were sacrificed after photodynamic therapy. Tumors and major organs (heart, liver, spleen, lung, and kidney) were collected and kept in 4% formalin followed by embedded in paraffin. Slices cut from the paraffin sections were stained by hematoxylin and eosin (H&E) before scanned with a fluorescence microscope (Leica DM6000M). Blood of mice was collected intravenously post-injection with PBS and IPA/LDH at day 1 and day 7. A standard biochemical procedure was carried out for the separation and analyzation of blood.

### Statistical analysis

All the data were presented as means±s.d.

### Data availability

The data that support the findings of this study are available from the corresponding author on reasonable request.

## Electronic supplementary material

Supplementary Information
