# Supplementary material for: Nano-photosensitizer based on layered double hydroxide and isophthalic acid for singlet oxygenation and photodynamic therapy
Source: Nat Commun. 2018 Jul 18;9:2798. doi: 10.1038/s41467-018-05223-3 (PMC6052022; doi:10.1038/s41467-018-05223-3)
Supplement: Supplementary file 1 — Supplementary Information [file 41467_2018_5223_MOESM1_ESM.pdf]

Nano-photosensitizer based on layered double hydroxide and isophthalic acid for singlet oxygenation and photodynamic therapy

Gao *et al*

Supplementary Figures

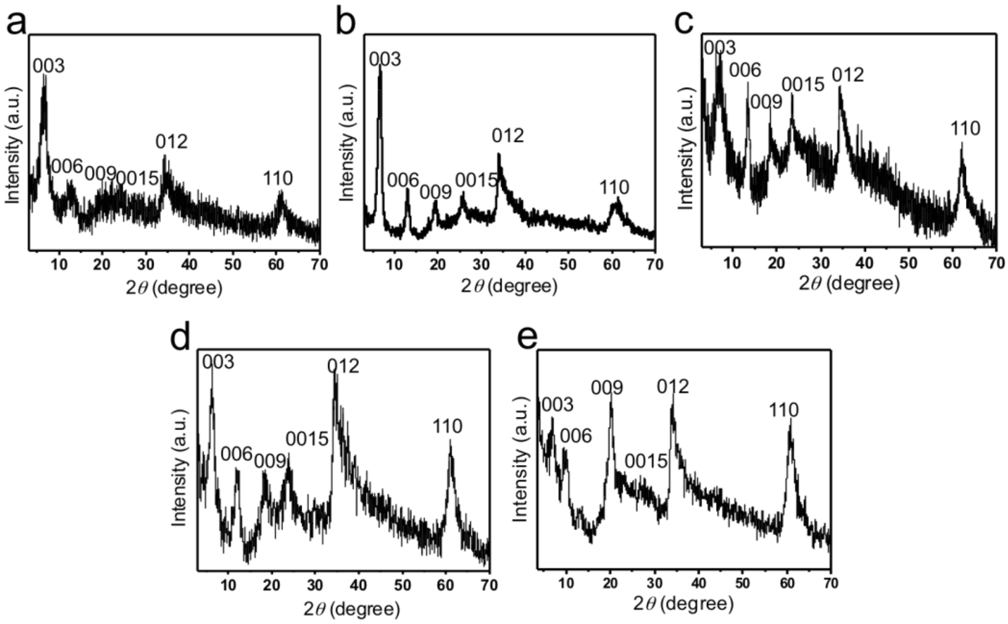

Supplementary Figure 1. XRD patterns. (a) BA/LDH, (b) IPA/LDH, (c) TA/LDH, (d) PMA/LDH, and (e) MA/LDH nanohybrids all exhibit typical reflections of LDHs structure.

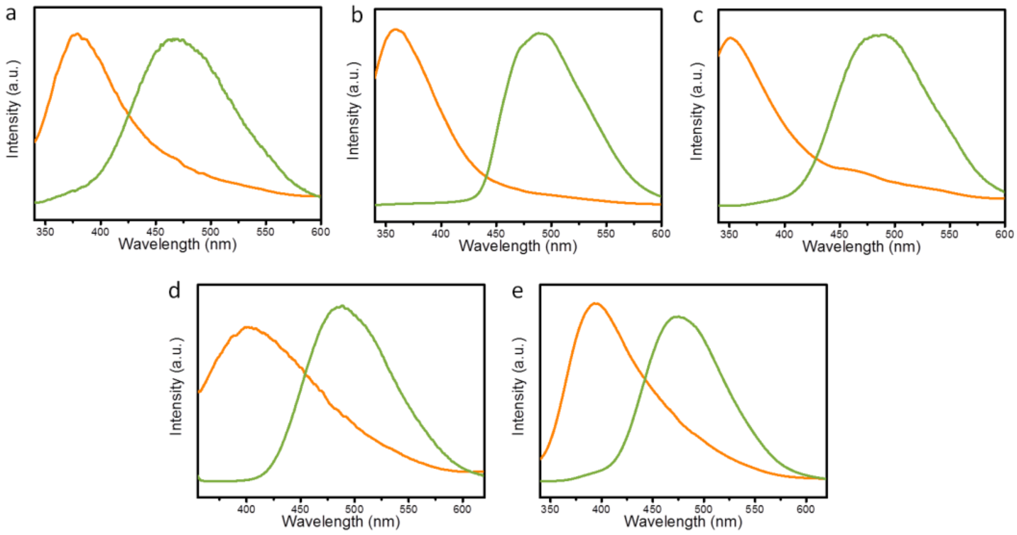

Supplementary Figure 2. Steady-state fluorescence (orange line) and phosphorescence (green line) characterization. Spectra of (a) BA/LDH, (b) IPA/LDH, (c) TA/LDH, (d) PMA/LDH, and (e) MA/LDH nanohybrids.

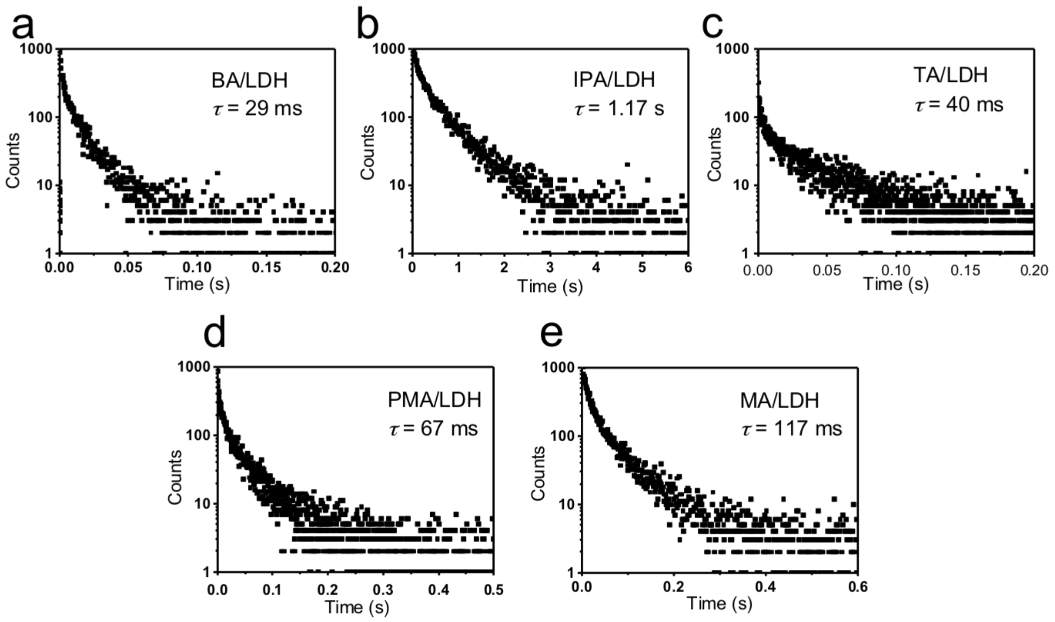

Supplementary Figure 3. Time-resolved emission decay profiles. The spectra of (a) BA/LDH, (b) IPA/LDH, (c) TA/LDH, (d) PMA/LDH, and (e) MA/LDH nanohybrids.

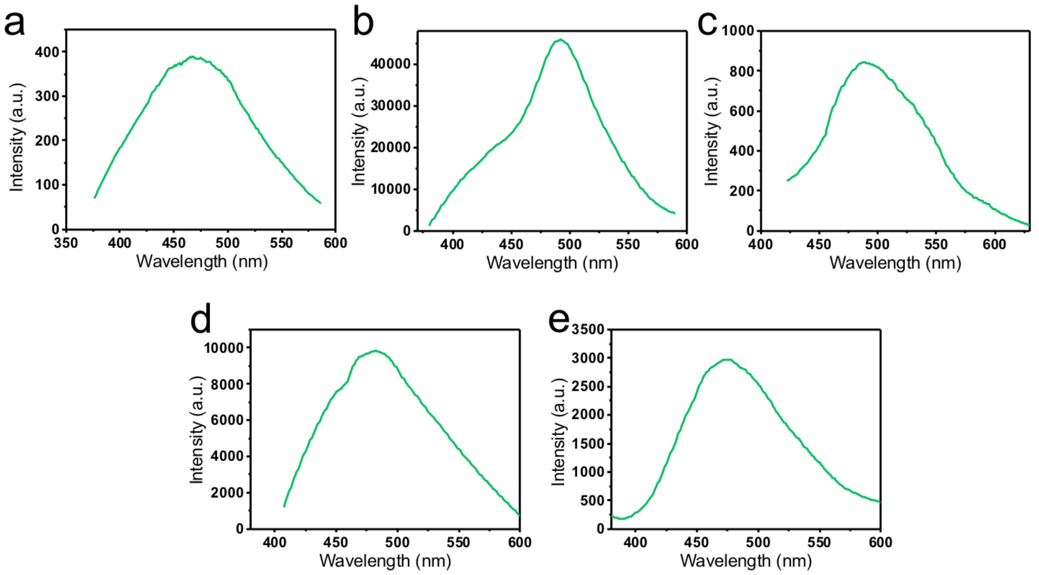

Supplementary Figure 4. Photoemission measurement. Spectra of (a) BA/LDH, (b) IPA/LDH, (c) TA/LDH, (d) PMA/LDH, and (e) MA/LDH nanohybrids excited by an 808 nm laser.

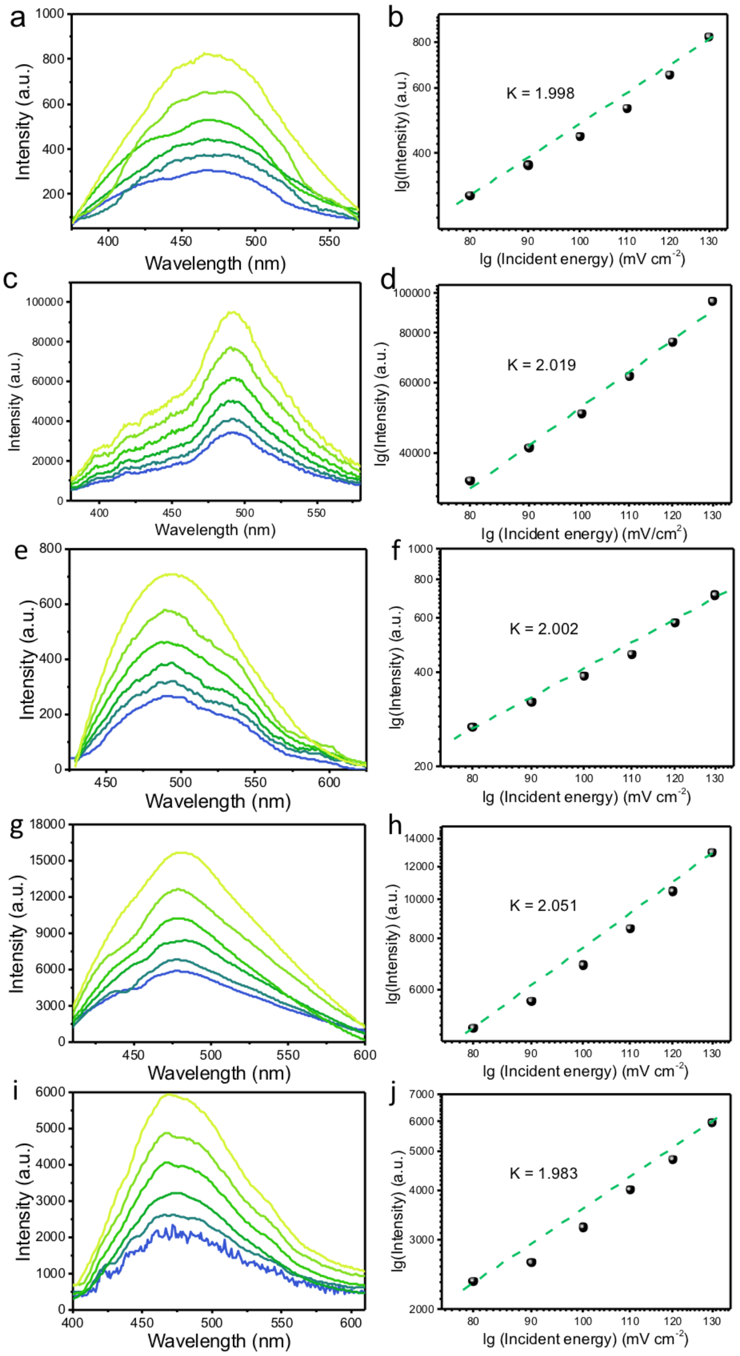

Supplementary Figure 5. Photoemission measurement with different incident powers. Spectra of (a) (b) BA/LDH, (c) (d) IPA/LDH, (e) (f) TA/LDH, (g) (h) PMA/LDH, and (i) (j) MA/LDH nanohybrids excited by an 808 nm laser under different incident powers.

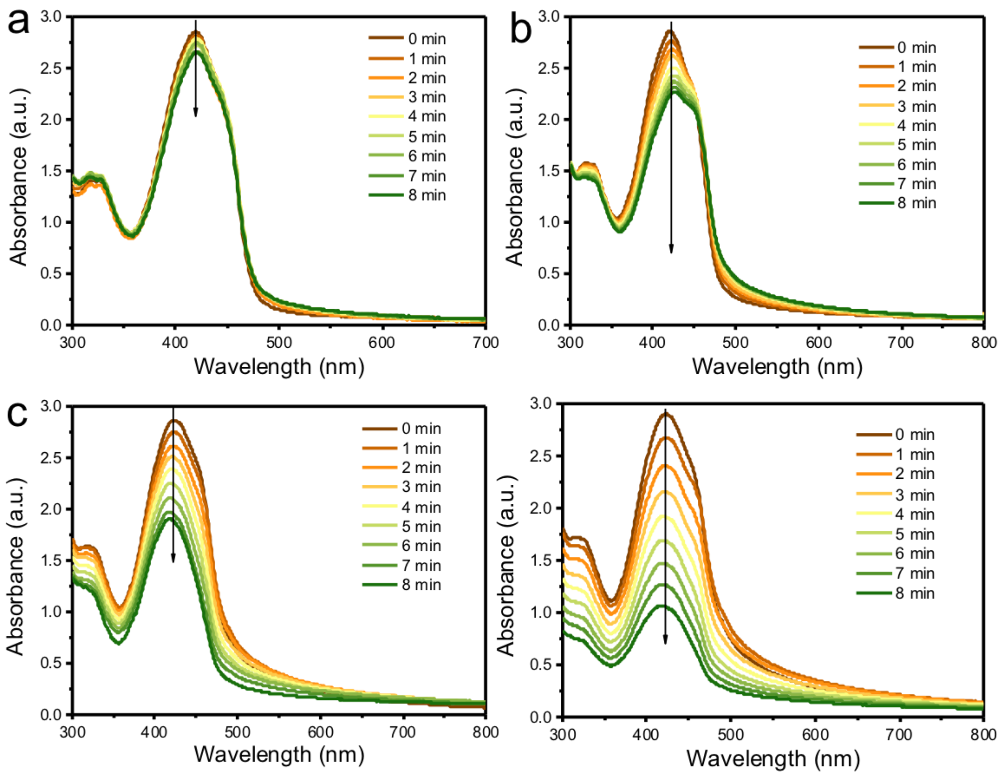

Supplementary Figure 6. Time-dependent absorption spectra of the DPBF. (a) Blank, (b) PMA/LDH, (c) MA/LDH, and (d) IPA/LDH nanohybrids in air.

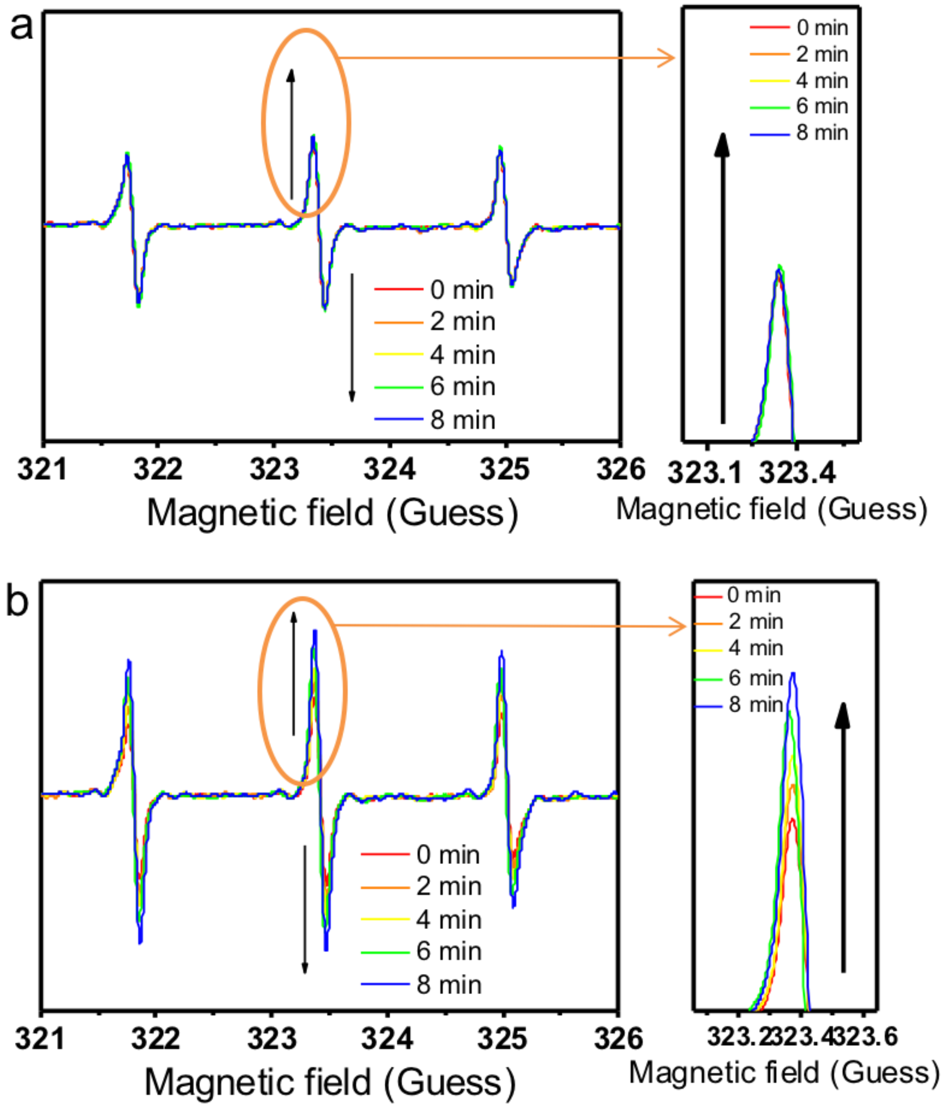

Supplementary Figure 7. Time-dependent ESR measurement. ESR Spectra of (a) PMA/LDH, and (b) MA/LDH nanohybrids in the presence of TEMP.

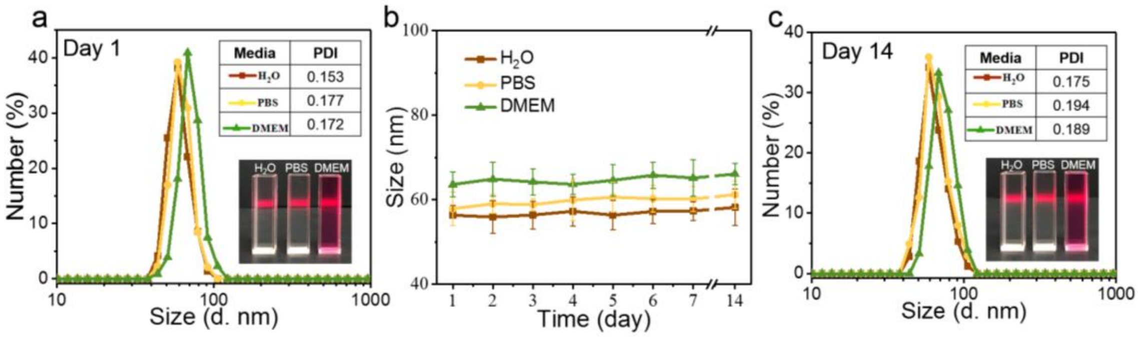

Supplementary Figure 8. Stability of IPA/LDH in different mediums. Size distribution of IPA/LDH in water, PBS and DMEM culture medium at (a) day 1 and (c) day 14. Inset: corresponding polydispersity indexes (PDI) and images of Tyndall effect. (b) Hydrodynamic diameter of IPA/LDH in water, PBS or DMEM for 14 days. Error bars were defined as standard deviation, *n* = 3.

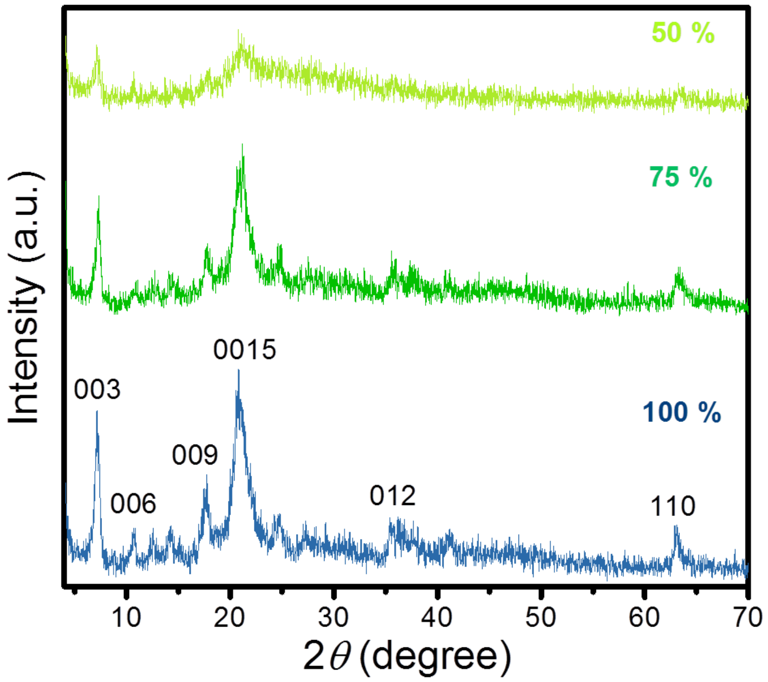

Supplementary Figure 9. XRD patterns of 50%-IPA/LDH, 75%-IPA/LDH, and 100%-IPA/LDH.

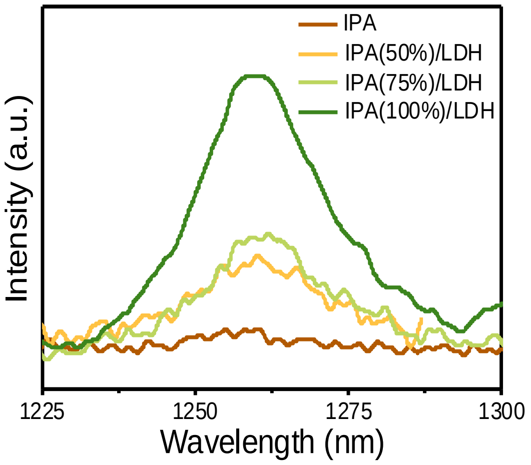

Supplementary Figure 10. ${}^{1}O_2$ emission at around 1270 nm induced by IPA(x%)/LDH and pure IPA in water.

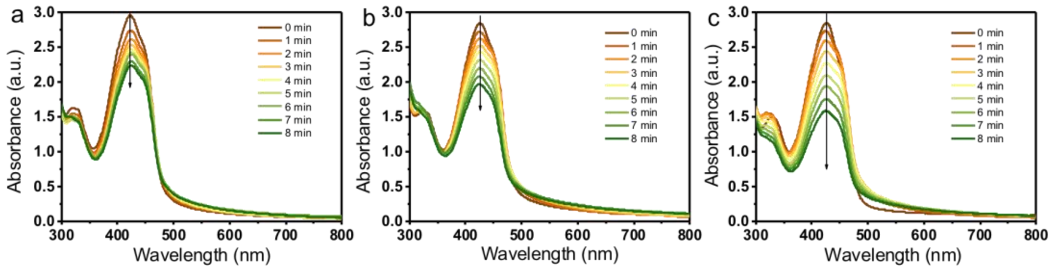

Supplementary Figure 11. Time-dependent absorption spectra of the DPBF in different conditions. (a) IPA, (b) 50%-IPA/LDH, (c) 75%-IPA/LDH nanohybrids in air.

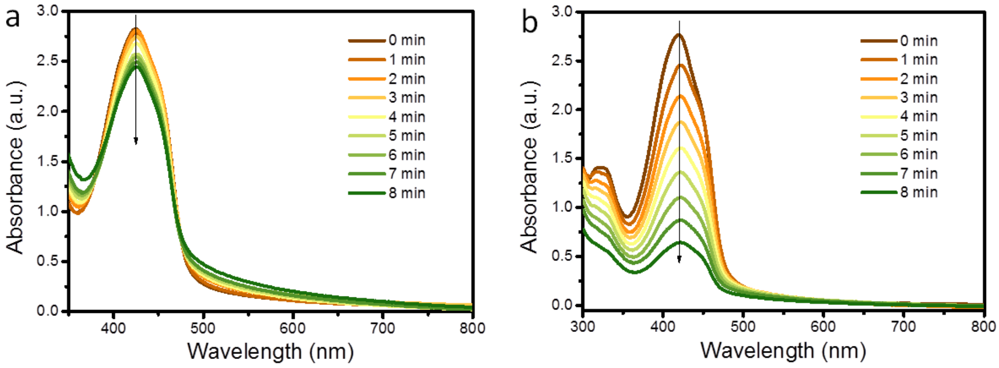

Supplementary Figure 12. Time-dependent absorption spectra of the DPBF in different conditions. (a) IPA/LDH nanohybrids in N_2 atmosphere; (b) IPA/LDH nanohybrids in O_2 atmosphere.

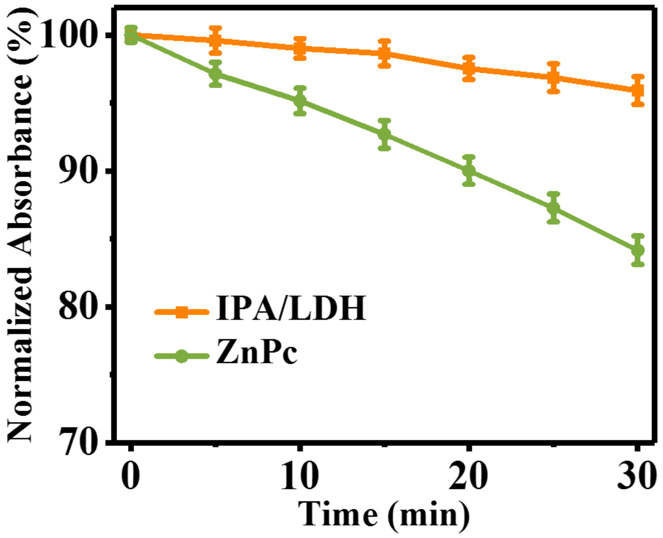

Supplementary Figure 13. Photostability of ZnPc and IPA/LDH under irradiation of a simulated sunlight source (100 mW cm^{-2}) for 30 min. Error bars were defined as standard deviation, n = 3.

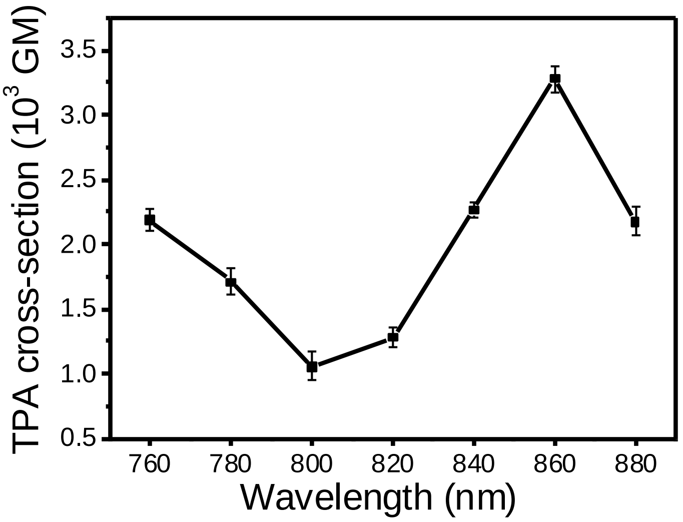

Supplementary Figure 14. Two-photon absorption cross section of IPA/LDH at different wavelengths. Error bars were defined as standard deviation, n = 3.

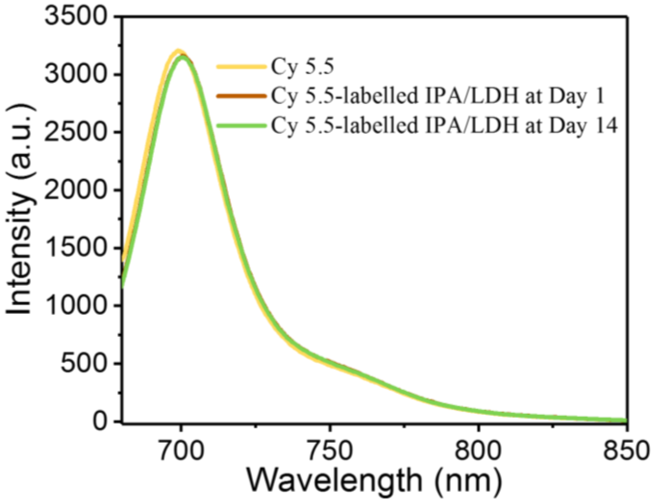

Supplementary Figure 15. Fluorescence emission spectra of the Cy5.5 and Cy5.5-labelled IPA/LDH storage in PBS at day 1 and day 14 (λ_{ex} = 675 nm).

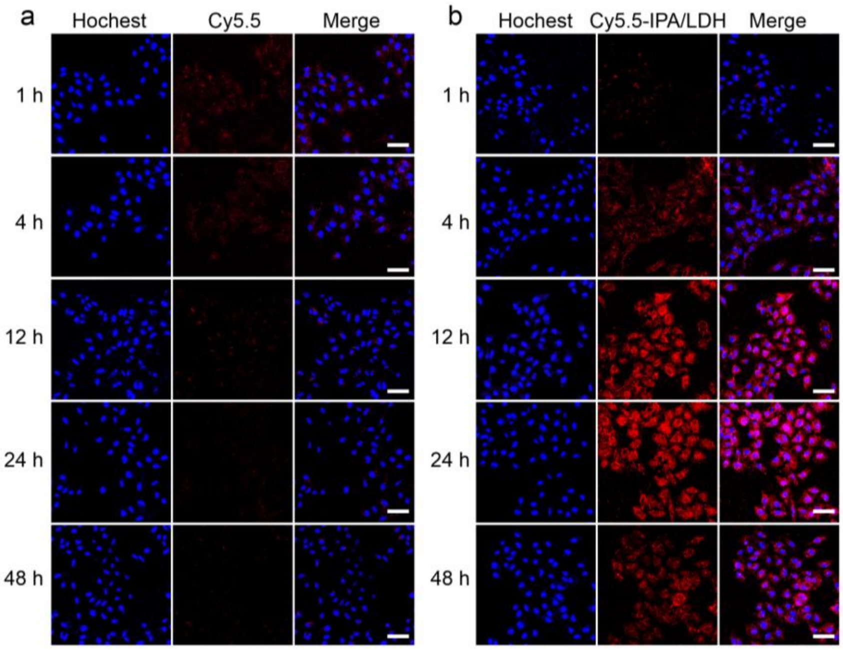

Supplementary Figure 16. Cellular uptake of IPA/LDH. Hela cell incubated with (a) Cy5.5 and (b) Cy5.5 labeled-IPA/LDH for 1 h, 4 h, 12 h, 24 h and 48 h, imaged by confocal microscopy. Nuclei were stained by Hochest 33342 (blue). Scale bar, 50 μm.

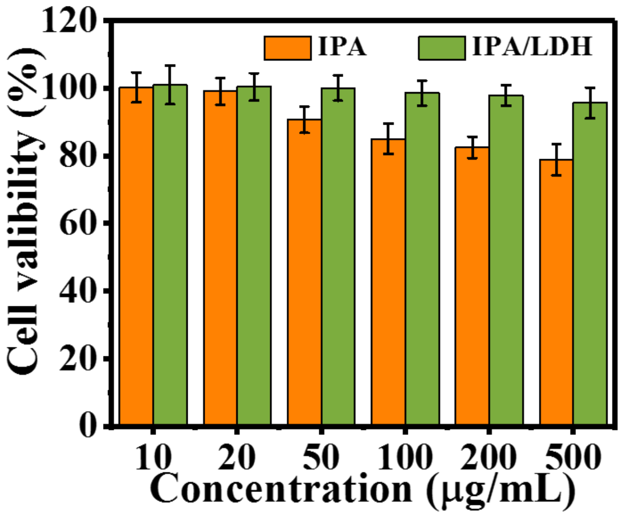

Supplementary Figure 17. Cell viability of cells incubated with various concentrations of IPA and IPA/LDH. Error bars were defined as standard deviation, *n* = 3.

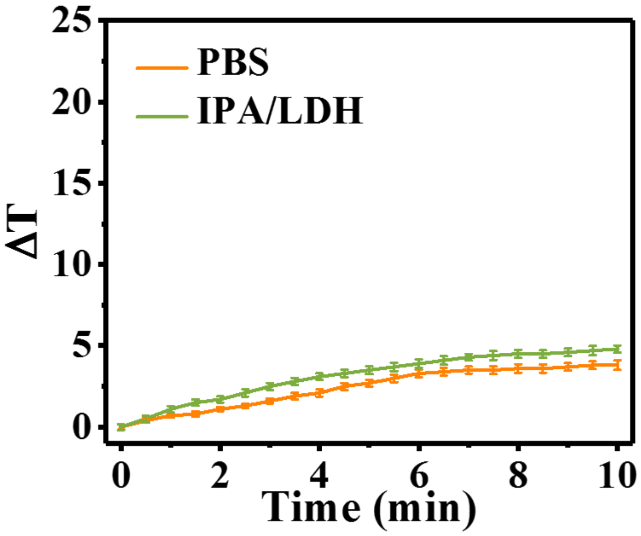

Supplementary Figure 18. Photothermal effect of IPA/LDH and PBS. Error bars were defined as standard deviation, *n* = 3.

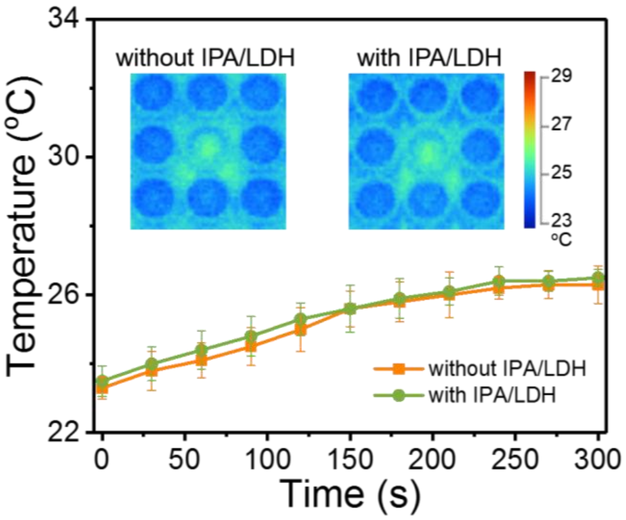

Supplementary Figure 19. Temperature elevation of cells. The temperature of cells incubated with or without IPA/LDH as a function of laser irradiating time (808 nm, 1.0 W cm^{-2}). Inset: Infrared thermographic maps of cells after laser irradiation for 5 min. Error bars were defined as standard deviation, n = 3.

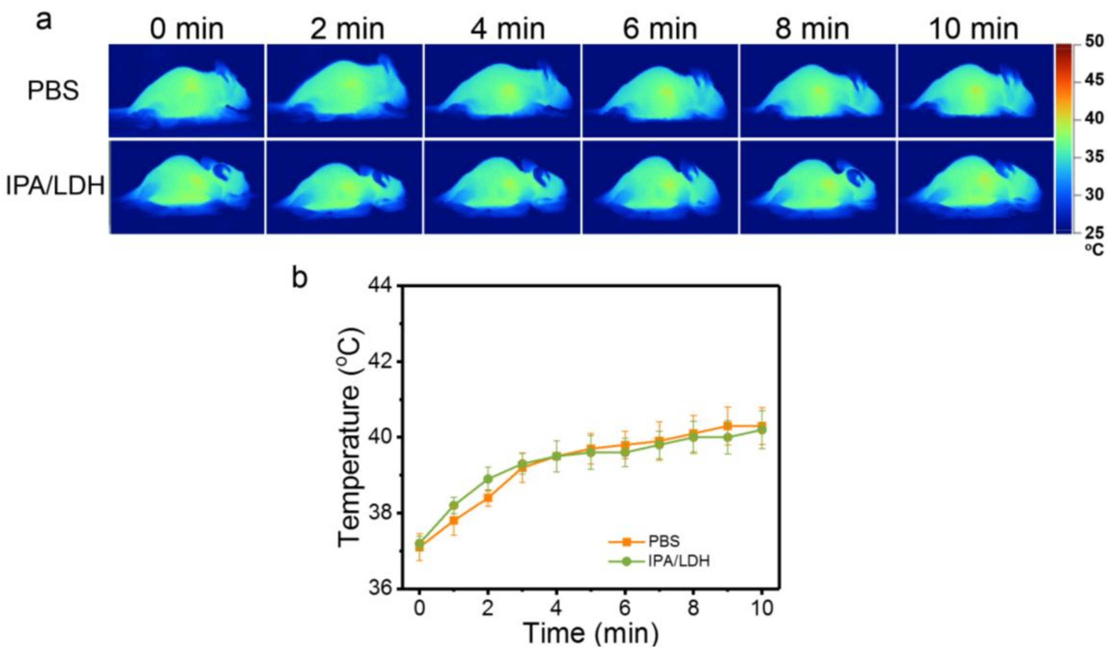

Supplementary Figure 20. Temperature elevation of mice. (a) Infrared thermographic maps and (b) Time-dependent temperature increase in the Hela tumour-bearing nude mice irradiated by the 808nm laser (0.7 W cm^{-2}) at 12 h after separated intravenous injection with 200 μL of PBS and IPA/LDH. Error bars were defined as standard deviation, n = 3.

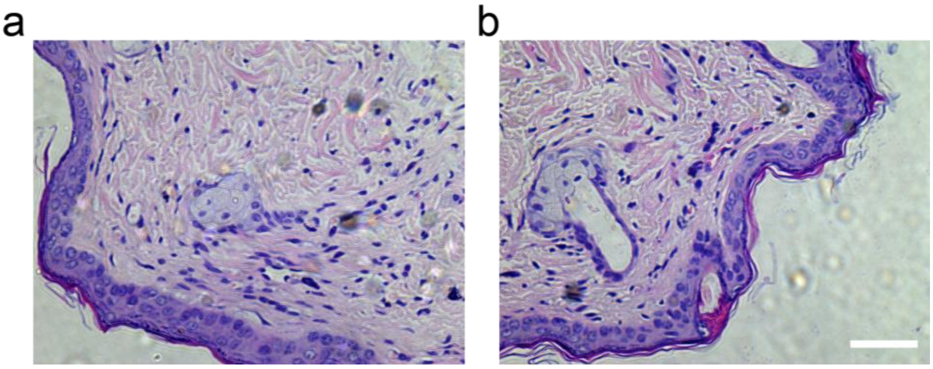

Supplementary Figure 21. No evident skin damage is observed with irradiation *in vivo*. H&E staining of skin collected from mice after *i.v.* injection of IPA/LDH (a) before and (b) after irradiation (808 nm, 0.7 W cm^{-2}) for 10 min. Scale bar, 40 μm.

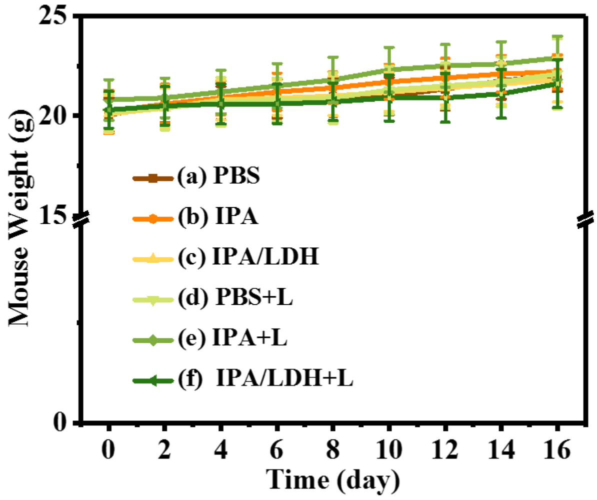

Supplementary Figure 22. The body weight changes of Hela tumor bearing mice after different treatments. Error bars were defined as standard deviation, n = 6.

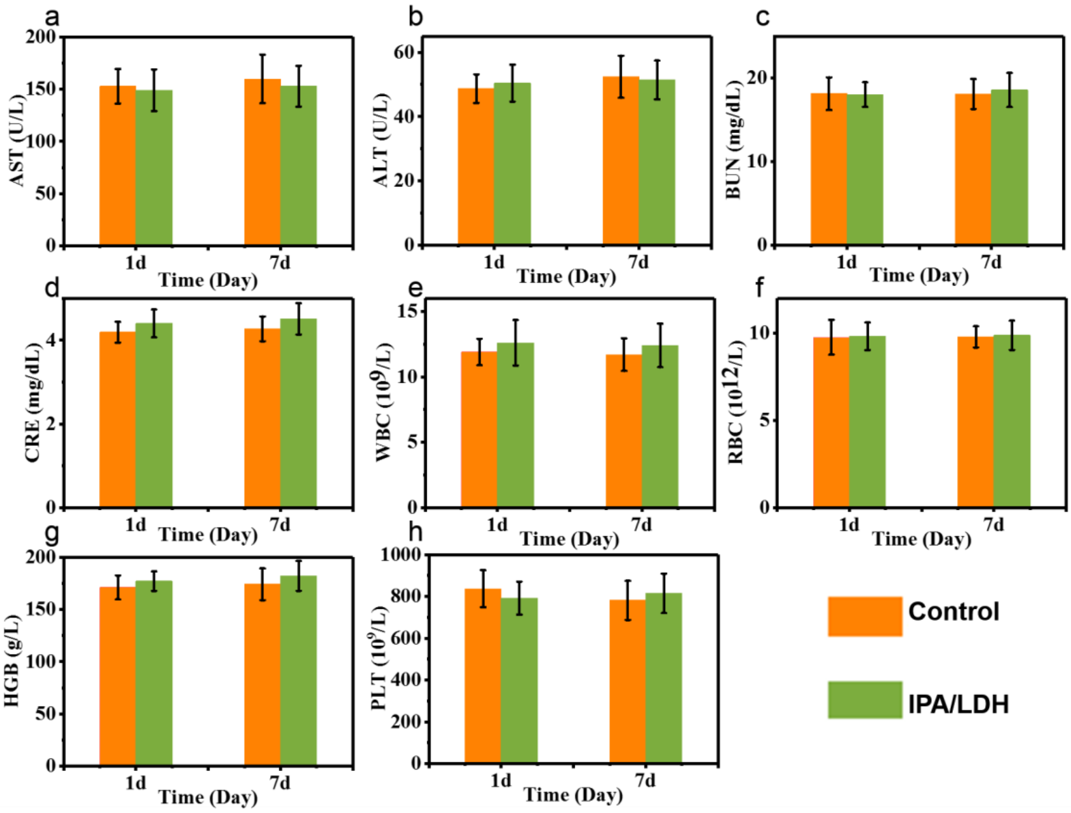

Supplementary Figure 23. *In vivo* toxicity. Liver and kidney function markers of (a) aspartate aminotransferase (AST), (b) alanine aminotransferase (ALT), (c) blood urea nitrogen (BUN), (d) creatinine (CRE); and blood biochemistry data including (e) white blood cell (WBC) counts, (f) red blood cell (RBC) counts, (g) hemoglobin (HGB), (h) platelets (PLT) for control group and IPA/LDH group after 1 and 7 days. Error bars were defined as standard deviation, $n = 3$.

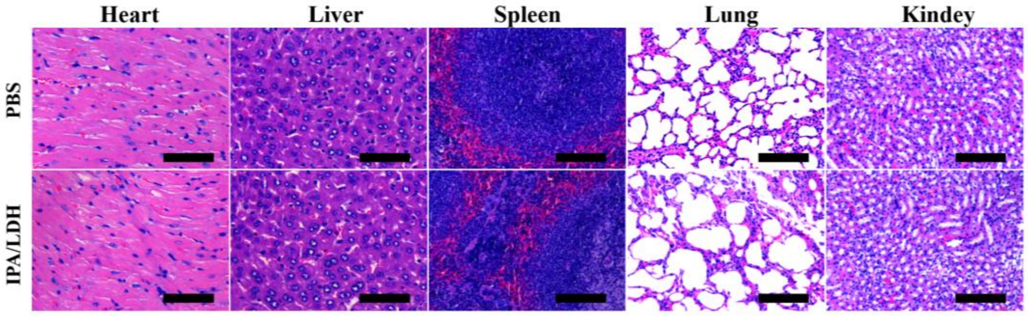

Supplementary Figure 24. H&E images of the major organs treated with PBS and IPA/LDH and collected on day 16. Scale bar, 100 μm.
